# Pharmacological labour pain interventions: South African midwives’ perspective

**DOI:** 10.1186/s12912-024-01844-w

**Published:** 2024-03-14

**Authors:** LE. Parkies, D. Murray, U. B. Okafor

**Affiliations:** 1 Department of Public Health, University of Fort Hare, 5 Oxford Street, 5201 East London, South Africa; 2 Department of Nursing Science, University of Fort Hare, 50 Church Street, 5201 East London, South Africa; 3 Faculty of Health Sciences, University of Fort Hare, 5 Oxford Street, 5201 East London, South Africa

**Keywords:** Midwife, Labour pain, Pharmacological relief pain, Interventions

## Abstract

Women of childbearing age feel great about giving birth, but the pain could be excruciating depending on their pain tolerances. Midwives requires obstetrical knowledge and skills such as pain management during labour and safety. We explored midwives’ perspectives on the utilisation of pharmacological pain alleviation interventions during labour in selected hospitals in Matjhabeng Municipality, Free State province, South Africa. A qualitative study was undertaken, involving a sample of ten midwives, using a semi-structured interview guide. The interviews were audio-recorded and transcribed verbatim. Tesch’s open coding data analysis method was applied to analyse the data. The midwives were restricted to use Pethidine and Phenergan prescribed by doctors for labour pain relief, which disrupted labour pain management and obliged them either to wait for a physician or follow telephone instructions. According to the midwives, women taking Pethidine and Phenergan encountered adverse effects and discomfort. Midwives identified high workload, inadequate personnel, lack of skill and knowledge, lack of medication availability, and lack of infrastructure as the primary challenges of administering pharmacological methods to women in labour. The lack of standing orders, which delays the administration of medications pending a physician’s prescription, constituted an additional difficulty. In the instance that Pethidine and Phenergan were unavailable or ineffective for some women, the midwives recommended that women be administered alternative pharmacological pain relievers. They also advocated for institutionalization of pharmacological guidelines allowing them to use their discretion when treating labour pain. Midwives can only utilise a few standardised and regulated pharmacological medications for labour pain management. The midwives’ ability to administer pharmacological pain relief during labour was hampered by a high workload burden, insufficient staff, lack of skill and understanding, drug unavailability, and inadequate infrastructure. Midwives advocated for supported guidelines that would allow them to treat labour pain at their discretion. Intersectoral stakeholders are required to improve midwife skills and attitudes. Health facilities need to train and supply analgesics to midwives. Midwives ought to be familiar with pharmacological pain relievers.

**Supplementary Information:**

The online version contains supplementary material available at 10.1186/s12912-024-01844-w.

## Introduction

Childbirth is an extraordinary event in a woman’s life that is typically accompanied by excruciating pain. However, the intensity of labour pain differs among women due to a combination of context-specific factors, including sociocultural, labour-related fear and anxiety, first or subsequent labour, environmental, and woman’s specific underlying disease or pathology. The WHO [1] has deemphasised the need for an exclusive focus on pharmacological techniques for assisting women with labour pain relief, instead encouraging and supporting women to engage in non-pharmacological strategies that are simple, inexpensive, and relatively safe.

The most common pharmacological strategy used to manage labour pain is the administration of epidural opioids [2]. This strategy is popular because of its widespread availability, ease of use, and low cost. Notwithstanding the advantage of opioids in producing analgesia with mild effect on sensation and proprioception [2], their negative side-effects during labour include nausea, vomiting, pruritus, sedation, and respiratory depression [3–5]. Furthermore, drug labour pain relievers include inhaled nitrous oxide (N2O) as an old-age analgesia pain relief during labour [6], meperidine [2], nalbuphine [7], and morphine [8]. Tramadol [9, 10], fentanyl [8], and remifentanil patient-controlled analgesia [11] are also utilised as labour pain relievers. Acetaminophen has been shown to be the safest, requiring minimal monitoring [12, 13], providing modest pain relief [14], and having fewer maternal side effects [11, 15]. The aforementioned pharmaceutical techniques for reducing labour pain have significant side effects as well can be costly [2, 16]. Given the negative effects and high cost of pharmacological therapies for labour pain management, it is critical to explore and utilise alternative methods of reducing labour pain. The intense pain associated with labour demands a significant requirement for healthcare personnel to aid women in relieving their labour pains. As a result, the challenge is for midwives and nurses to live up to their obligations by ensuring a less painful delivery experience. The best strategy would be to actively involve women in the birthing process by offering supportive measures based on humanistic values (autonomy, empathy, respect for women’s and families’ rights), rather than recourse to pharmacological therapies.

In South Africa, as in other nations, sedatives, hypnosis, and tranquillizers are recommended and used therapeutic measures during labour, with Pethidine and Phenergan being the most frequently prescribed medications [17–19]. The administration of these drugs is either intramuscular or intravenous routes, serving as systemic analgesics [20, 21]. In addition, midwives in South Africa adapt pain-relieving medications based on their own volition and a physician’s recommendation [17, 19]. It should be noted that contextual factors may influence the decision of midwives regarding labour pain relief, which may be based on the midwife’s expertise and understanding of health policy or guidelines, and health system challenges operating in various contexts. Regarding what, when, and how to administer pharmacological labour relief methods, midwives may find these concerns challenging due to a lack of knowledge and training on pain-relieving pharmacological strategies. Understanding the perspectives of midwives on pharmacological labour pain management is crucial to enhancing their judgment and decision-making regarding the provision of palliative labour pain treatment to their clients in such scenarios. Despite this, little is known about midwives’ perspectives on the utilisation of pharmacological interventions for labour pain interventions in clinical health settings in South Africa. Therefore, this study explored the experiences of midwives concerning the utilisation of pharmacological labour pain strategies in clinical practise in Matjhabeng Municipality hospitals of the Free State province, South Africa.

## Materials and methods

### Research design and participants

A qualitative, descriptive, exploratory, and contextual study approach was utilised to gain insight about midwives’ perspectives on pharmaceutical labour pain management methods in the Lejweleputswa District. Qualitative research seeks to address questions about participants’ real-life experiences, meanings, and perspectives (Hammarberg, Kirkman, & de Lacey 2016). Descriptively, understanding midwives’ experiences with pharmacological labour pain management interventions would eventually lead to an exploration of the relatively new phenomenon of labour pain management techniques among midwives working in maternity settings in Matjhabeng Municipality, Lejweleputswa District, Free State province. Such a design approach could improve our understanding of pharmaceutical methods in managing birth pain relief in this setting, as well as laying the groundwork for future research on the subject.

A purposive sample of midwives employed in the maternity wards of two conveniently selected hospitals in Matjhabeng Municipality, Lejweleputswa district, served as the participants of this study. An in-person interview with a semi-structured format was conducted. Participants were selected based on the following criteria: age between 28 and 55, minimum of 3 years of work experience, and willingness to sign the informed consent form.

### Setting

The study was conducted in the maternity wards of two Matjhabeng Municipality, Lejweleputswa District, Free State-run health facilities with scarce resources. According to Statistics South Africa’s 2016 Census, Matjhabeng has an estimated population of 42,913 people [22]. The municipality contains three district hospitals, one regional hospital, three private hospitals, and day facilities that do not offer intrapartum care unless it is an emergency. These hospitals offer hybrid maternity facilities, with a maximum of 11 midwives in hospital A and 15 midwives in hospital B, respectively.

### Data collection procedure

Participants who agreed to participate were requested to submit their signed consent forms to the principal investigator (LEP). They were then informed through phone calls about the interview date, time, and location. The research tool applied a semi-structured interview guide with broad questions regarding the use, challenges, and suggestions for enhancing pharmacological labour pain (Supplementary file 1). A total of ten participants were interviewed in-person to obtain data. The interviews were audio-recorded with the participants’ approval, and each session lasted approximately 40–60 min.

The study’s trustworthiness was ensured using a variety of methods. LEP performed member checking, audio trials, and multiple double-checking of the transcribed data. A cordial relationship was built with the participants by proper description of the research objectives, and sufficient time was spent interviewing them to guarantee long-term commitment. In addition, interviews, field notes, and an audio recorder were used to collect important information from participants. By maintaining an audit trail, the raw data was preserved from each interview for future reference. To ensure the study’s transferability, all data transcripts, audio recordings, and independent coder analysis were meticulously maintained. Furthermore, the second author (MD) scrutinised the analysed data, and peer-reviewed it to ensure that the results reflected the participants’ voices. In addition, the interview transcripts and emerging themes were validated by the participants. Lastly, an independent coder verified the data, and the respondent’s approved transcriptions and themes.

### Data analysis

The Tesch’s eight-step coding process for qualitative data analysis was used to analyse the data as described by Creswell [23]. The entire transcript was carefully studied to get a sense of the whole, taking brief and important notes. Then, a case-by-case approach was used to discern the underlying meaning in the material, which was penciled down in the margin. Following that, a list of all the themes or topics was collected, and comparable themes or topics were clustered together before being applied to the data, abbreviated as codes, and written next to the appropriate segments of the transcripts. Furthermore, the most descriptive wording for the themes or topics was classified; and then, lines were drawn across categories to demonstrate relationships. A final selection on the abbreviation for each category was reached, and the codes were organised alphabetically, as well as the data material associated with each category, and preliminary analysis was undertaken. Lastly, existing material was recoded as needed. Principal investigator (LEP) and independent coder determined the main theme and sub-themes.

### Ethics statement

The University of Fort Hare’s Health Sciences Ethics Research Committee approved the study’s ethics (Reference number: 2021 = 05 = 02 = ParkiesL). In addition, the nature and purpose of the study were conveyed to the participants, who provided their written consent after being fully informed.

## Results

Participants were between 28 and 55 years. Most midwives had vast professional experience in midwifery. Themes and sub-themes identified focused on the participants’ positive and negative experiences, challenges, and suggestions for improving pharmacological labour pain reduction strategies.

### Theme 1: experiences applying pharmacological interventions

#### Effects on labouring women

Midwives in hospital maternity wards use pharmacological pain management techniques to alleviate labour pain. Most participants reported that Pethidine and Phenergan effectively sedate, calm, and alleviate labour pain in women.

*“It’s pretty good. Most of the time, the results are good since, once administered, it relieves pain and allows the patient to sleep and rest, but not always”* (MW 7).

*“I’ve noticed that giving sedation to patients allows them to rest and reduces their pains”* (MW 6).

*“The sedation is effective to some, I say so because the women will be screaming during a contraction but after sedation, they become calm and stop screaming” (*MW 3).

### Theme 2: positive experiences of pharmacological interventions

To promote greater effectiveness in labour processes and a satisfying delivery, midwives routinely gave women pharmacological pain relievers to all women during the childbirth process.

#### Useful and good intervention

Some participant experiences indicate that Pethidine and Phenergan aid in the rapid dilatation of the cervix, thereby expediting childbirth.

*“But what I do know is that it has an effect on the cervix, as the woman is likely to relax and give birth in a matter of minutes”* (MW 2).

*“I have realised that Phenergan actually speeds up the process of labour. If a woman is 1 centimetre dilated and a primigravida, and you give her this combination of Phenergan and Pethidine, within four hours she is fully dilated, bearing down well, and giving birth without any difficulty”* (MW 5).

*“Once you have administered sedation, the pain subsides for the reason the patient may sleep, rest, and then dilate more quickly and give birth without complication”* (MW 7).

Some participants indicated that Pethidine and Phenergan have advantages since they relax women, causing them to cooperate with the goal of preventing labour complications.

*“Pharmacological labour pain management I believe it is really useful and helpful, especially for women in labour, since if you sedate your patient, the patient will benefit and be cooperative, preventing complications”* (MW 3).

*“It works for most women, because you will find that the woman is relaxed”* (MW 9).

*“We give even at 8 cm; 9 cm we can still give so that she can be relaxed”* (MW 8).

*“The effect of this pharmacological pain management on pregnant women is quite good because it helps the woman to relax”* (MW 7).

Based on the midwives’ experiences, Pethidine and Phenergan are effective pharmacological labour pain treatment approaches.

### Theme 3: negative experiences of pharmacological interventions

Participants expressed Pethidine and Phenergan had no effect on some labouring patients, who continued to complain of pain despite receiving these medications.

#### Sometimes effective medications: pain persists

Some participants reported that Pethidine and Phenergan had no effect in certain women, as they continued to scream after being sedated, while others requested additional dosages.

*“In other situations, some women continue to scream and sob even after you’ve sedated them, and they’ll then want the medication again since they believe it didn’t work on them”* (MW 1).

*“My experience here is that the patients are not all experiencing the same effects; for some, pharmacological sedation has an effect, while for others, it does not. For some patients, the effects are mixed. The patients are not all the same because some of them are still in pain after receiving a sedative”* (MW 4).

*“Sometimes with other patients it doesn’t really work because even after you give them the sedation, they are still having the pains”* (MW 6).

### Theme 4: challenges: pharmacological interventions

Midwives face challenges while using pharmacological pain treatment approaches, which disrupt the pain management regimen. The health care system and the detrimental effects of the medications on the mother and infant pose challenges.

#### Absence of guidelines governing usage of drugs

One respondent indicates the absence of regulatory guidelines on pharmacological pain treatment measures, which necessitates regular approval from doctors, potentially leading to harmful outcomes.

*“We do not have standing orders like other institutions where there is a standing order that you should sedate, thus you must always call the doctor to get permission to sedate the patient”* (MW 3).

Standing orders instructing midwives on when and how to administer painkillers are not always available, making it challenging to carry out a pain management schedule and causing suffering in women who are awaiting a prescription from a doctor.

#### Maternal

Most participants stated that the use of Pethidine and Phenergan have adverse effects on the mother. Drowsiness, exhaustion, sleepiness, and a lack of energy are a few of these, which impede women from pushing at the scheduled time.

*“Sometimes the mother will be tired and sleepy, even though she’s fully dilated, the baby’s head will be crowning, and you will be telling her, push the baby out, she will be just sleeping and saying sister what are you saying, I don’t have the energy to do that”* (MW 1).

*“The problem becomes significant because the sedation is so high, and they become sleepy.”* (MW 5).

*“Sometimes you give the patient the sedation during an active phase of labour and when is time for delivery, they are unable to push the baby out because they are still drowsy”* (MW 6).

Some participants report that these medications are occasionally out of stock, forcing them to rely only on non-pharmacological approaches.

*“Occasionally, these drugs are out of stock, so women do not receive them as they should; therefore, in this instance, we only use non-pharmacological interventions”* (MW 7).

*“The challenge is that sometimes they are out of stock”* (MW 8).

Because Pethidine and Phenergan are not readily available in birth wards, midwives are unable to control pain as planned. Women must merely accept the pain that ensues, which has severe consequences such as early bearing down, resulting in newborns with low Apgar scores.

#### Foetal

Most participants indicate that some babies are born floppy because of the moms’ drowsiness during the active period of labour. This suggests that these medications can cross the placenta, which explains the floppiness. They further highlighted that pharmacological pain therapies have unanticipated implications for some newborns who are born with respiratory depression and low Apgar scores.

*“Some babies are floppily when they are born because of the sedation”* (MW 2, 10).

*“The problem is that the baby will appear floppy after it is born due to the sedative’s effects”* (MW 7).

*“Some of the babies will be having respiratory distress, but not many”* (MW 4).

### Theme 5: suggestions for improvement

Midwives made recommendations concerning use of pharmacological methods include alternative painkillers if the preferred opioids are out of stock or if the medications do not work for some women.

#### Use of “love gas”

Some participants propose that other pain-relieving methods, such as gas and air, should be explored because they are also effective in alleviating labour pain.

*“I think they can use other pain analgesics may be something that is stronger for pain besides the sedation and besides the injection, may be the oral medication, especially the primigravida when they are in latent phase”* (MW 1).

*“So, if they can explore those others like the gas that is now been used, they call it loving gas I don’t know what its name is”* (MW 7).

#### Formulate regulatory protocols

Some participants recommend the need for standing orders to guide midwives on the use of pain management drugs in the absence of the doctor.

*“I feel it is preferable if we can have a standing order that can give us, perhaps permission to say if there are no complications or is just a pain, we can sedate the patient rather than calling the doctor first. Okay, you can still call but the standing order must be there”* (MW 3).

## Discussion

This study explored the perspectives of midwives in Matjhabeng Municipality, Free State Province, South Africa, about the usage of pharmacological labour pain therapies in hospital settings. Doctor-prescribed Pethidine and Phenergan were the only major pharmacological pain treatment choices available to labouring women, which disrupted labour pain management because midwives had to wait for the doctor or follow phone instructions. Even though these drugs promote cervical dilation and shorten labour, the women experienced discomfort while taking them. The deleterious effects on the mother and infant, as well as the advancement of labour, are difficulties resulting from the administration of these drugs. In addition, palliative medications are not always available, which can cause emotional discomfort. The midwives advocated for the use of additional non-pharmacological therapies, such as love gas, and stressed the need for guidelines that will enable them to exercise their discretion in treating labour discomfort.

Systemic opioids (Pethidine and Phenergan) are the sole main pharmacological labour pain strategies employed by midwives in this study, maybe due to a lack of any other procedures or departmental policies, as these medications are recommended by the doctor. However, the procedures stated above disrupt labour pain management, requiring midwives to either wait for the doctor or follow phone instructions. Both Pethidine and Phenergan work effective in sedating and calming the woman, as well as reducing birth pains [16, 17, 24, 25]. Like our study, other studies have reported the use of pethidine and Phenergan as analgesics in various African countries, which include Egypt [26], Ghana [27], Ethiopia [24, 28], and Nigeria [29]. The use of pethidine during labour, either intramuscularly (IM) or intravenously (IV), at low doses of up to 50 mg has been shown to be safe for the foetus, and its administration to women during childbirth has been associated with Apgar scores of 7 or higher, normal pH and arterial blood gases in infants, and no respiratory depression [30]. Clearly, the drug’s risk is dependent on its route of administration and dosage; therefore, the risk-benefit ratio of administering pethidine to women in labour should be considered [30]. Nonetheless, the of analgesics treatments may occasionally be ineffective in providing pain relief for all birthing moms following their administration; they are also prone to side effects for both the mother and the infant, and they might disrupt the progress of labour. According to studies, adverse side effects of analgesics during labour include nausea, vomiting, pruritus, sedation, and respiratory depression [3–5]. Notably, Pethidine is the most prevalent opioid used to alleviate birthing pain, especially in low-income and developing nations. Also, Pethidine remains pervasive across various obstetrics settings as it is inexpensive, readily available, and easy to administer, and because it is advantageous when alternative approaches are unavailable or contradicted [31]. Furthermore, these medication intervention approaches may not address the mother’s psychological and emotional well-being. Therefore, it is essential to explore non-pharmacological pain relief interventions that address the psychological and emotional well-being of the woman during childbirth.

Midwives stated that all women in labour receive Pethidine and Phenergan as standard care because this procedure improves cervical dilatation and shortens labour. Furthermore, because midwives do not emphasise the need for alternative pharmacological labour pain relief possibilities, implying their lack of awareness or concern about other accessible pharmacological labour pain relief options. This lack of awareness by midwives regarding other labour pain-relieving medications, restricts labour pain management alternatives and results in inadequate alleviation of pain, especially when medications are no longer in stock. The midwives were also constrained with the standard protocol established by the Department of Health, which recommended Pethidine and Phenergan as labour pain relieving therapies in healthcare facilities.

Midwives reported that despite the effectiveness of pharmacological approaches in reducing labour pains, some women experienced discomfort; women screamed and requested additional dosages. This could be attributed to the late administration of the pharmacological therapy or its ineffectiveness at relieving labour pain. High pain levels and frequent requests for more analgesia were reported by two groups of women during studies with Diamorphine and Pethidine, demonstrating that neither medication was effective [32].

Our findings revealed that the drawbacks of pharmacological pain management approaches include adverse consequences on women, labour progression, and newborns. In some cases, a paucity of palliative drugs created distress since midwives were compelled to adopt non-pharmacological measures that were not always effective. Midwives face difficulties such as the absence of standing orders, which delays the administration of medications pending a physician’s prescription [33]. Midwives noted maternal concerns about drowsiness, weariness, sleepiness, and a lack of energy, which influence the women’s ability to push during birth, as well as floppy neonates or respiratory distress due to sedative effect [34].

To mitigate the challenges of administering pharmacological interventions in clinical health settings, the midwives in this study advocated for the application of other pharmacological pain relievers that would help alleviate the labour pains of the women. This approach could be beneficial in situations where Pethidine and/or Phenergan are unavailable/ineffective for some women. According to Eyeberu et al. [28] study among obstetric care providers in Ethiopia, pethidine, diclofenac, and paracetamol were the primary three pain relievers administered to women during childbirth. The most frequently available medications were pethidine (68.9%), diclofenac (76.4%), paracetamol, and hyoscine. Similarly, Ghanaian midwives use paracetamol or other painkillers to alleviate childbirth pain [17], whereas British midwives apply nitrous oxide (gas and air) for labour pain analgesics [20]. In addition, midwives in our study advocated for standing orders and regulations that would allow them to use their discretion while treating labour pain.

Our finding is consistent with other studies that have identified high workload, inadequate staffing, lack of skill and knowledge, non-availability of medications, and lack of infrastructure as the primary barriers for administering pharmacological methods to women during labour [28, 35–37]. These findings suggest that access to maternal health services is impeded by a weak healthcare system, ineffective facility organisations, and inequitable or inefficient resource distribution [28]. However, the institutional availability of analgesics and equipment is substantially related to the use of labour pain management techniques [38]. As highlighted by the midwives in this study, providing in-service training to equip midwives with the relevant knowledge/skills and independent decision-making regarding the use of available pharmacological pain relief and equipment will facilitate the provision of pain relief to labouring women. These issues are particularly crucial in the context of this low-resource setting, which is characterised by an acute shortage of midwives and other obstetric care providers. The pain of labour is excruciating, and midwives are required to provide maternal care, which includes pain management during childbirth. This challenge becomes more inevitable when women demand that they require pain relief. There is a need for guidelines regulating the administration of pharmacological pain relief by midwives. This would mitigate the problem of waiting for the doctor’s prescriptions, as the doctor is occasionally unavailable. In turn, this will enable the midwives to apply their independent pain management skills.

## Limitations of the study

Given that this study was conducted in one geographic area, its results are unlikely to be generalised to other geographical contexts within the exact province or to various provinces. Also, we were also unable to explore the perspectives of women, or midwives at private health facilities; so, further study is required. This study emphasises the perspectives of midwives regarding the utilisation of pharmacological labour pain relief measures in this geographically-resource-constrained context, with policy and practise improvement implications.

## Conclusion

Midwives are restricted to a small number of standardised and regulated pharmacological pain relievers for labour pain management in the health facilities for which they are responsible, limiting the applicability of these medications and their autonomy in administering alternative pain therapies. The midwives advocated for the use of additional non-pharmacological therapies, such as love gas, and stressed the need for guidelines that will enable them to exercise their discretion in treating labour discomfort. To support and enhance the skills and attitudes of midwives, there is a need for the involvement of intersectoral stakeholders. The health care facilities should provide training for midwives to improve their skills while additionally supplying them with sufficient analgesics. Midwives also need to be knowledgeable about pharmacological pain treatment options.

### Electronic supplementary material

Below is the link to the electronic supplementary material.

Supplementary Material 1

## Data Availability

No datasets were generated or analysed during the current study.
