# Supplementary material for: Pharmacological labour pain interventions: South African midwives’ perspective
Source: BMC Nurs. 2024 Mar 14;23:176. doi: 10.1186/s12912-024-01844-w (PMC10938825; doi:10.1186/s12912-024-01844-w)
Supplement: Supplementary file 1 — Supplementary Material 1 [file 12912_2024_1844_MOESM1_ESM.pdf]

INTERVIEW GUIDE

SECTION A: DEMOGRAPHIC DATA

Name of the institution	
Participants No	
Age	
Work experience as professional nurse (in years)	
Work experience in midwifery (in years)	

SECTION B: INTERVIEW QUESTIONS

1.What are your experiences on use of pharmacological labour pain management interventions?<br><br>2.What are the challenges you experienced whilst using pharmacological pain management interventions?<br><br>3.What are your experiences on use of non- pharmacological labour pain management interventions?<br><br>4.What are the challenges you experienced whilst using non- pharmacological pain management interventions?<br><br>5.Tell me about what needs to be improved in labour pain management.
